# Fisher Discrimination Regularized Robust Coding Based on a Local Center for Tumor Classification

**DOI:** 10.1038/s41598-018-27364-7

**Published:** 2018-06-14

**Authors:** Weibiao Li, Bo Liao, Wen Zhu, Min Chen, Zejun Li, Xiaohui Wei, Lihong Peng, Guohua Huang, Lijun Cai, HaoWen Chen

**Affiliations:** 1 College of Information Science and Engineering, Hunan University, Changsha, Hunan 410082 China; 2 Hunan University of Technology, Zhu Zhou, Hunan 412007 China

## Abstract

Tumor classification is crucial to the clinical diagnosis and proper treatment of cancers. In recent years, sparse representation-based classifier (SRC) has been proposed for tumor classification. The employed dictionary plays an important role in sparse representation-based or sparse coding-based classification. However, sparse representation-based tumor classification models have not used the employed dictionary, thereby limiting their performance. Furthermore, this sparse representation model assumes that the coding residual follows a Gaussian or Laplacian distribution, which may not effectively describe the coding residual in practical tumor classification. In the present study, we formulated a novel effective cancer classification technique, namely, Fisher discrimination regularized robust coding (FDRRC), by combining the Fisher discrimination dictionary learning method with the regularized robust coding (RRC) model, which searches for a maximum a posteriori solution to coding problems by assuming that the coding residual and representation coefficient are independent and identically distributed. The proposed FDRRC model is extensively evaluated on various tumor datasets and shows superior performance compared with various state-of-the-art tumor classification methods in a variety of classification tasks.

## Introduction

Microarray techniques have been used to delineate cancer groups or to identify candidate genes for cancer prognosis. The accurate classification of tumors is important for cancer treatment. With the advancement of DNA microarray and next-generation sequencing technology^1–4^, various gene expression profile (GEP) data are rapidly obtained. Thus, we should develop novel analysis methods that can deeply mine and interpret these data to obtain insight into the mechanisms of tumor development. To date, a number of methods have been proposed for classifying cancer types or subtypes^5–9^. These common methods, including support vector machine^10^, linear discriminant analysis^11^, partial least squares (PLS)^12^, and artificial neural networks^13^, have been used to mine gene expression data.

Machine learning-based methods have been widely used in tumor classification. However, these methods require a predictive model to predict the labels of test samples. Predictive model selection is a complex training procedure that easily leads to overfitting and decreased prediction performance. Recently, given the non-requirement for model selection and robustness to noise, outliers, and incomplete measurements, sparse representation-based classifier (SRC) was proposed for face recognition^14,15^ and further extended to cancer classification^16–18^ and miRNA-disease association prediction^19,20^. For example, Hang *et al*. proposed a SRC-based method to classify six tumor gene expression datasets and obtained excellent performance^18^. Zheng *et al*. further combined the idea of metasample and proposed a new SRC-based method for tumor classification called metasample-based sparse representation-based classifier (MSRC)^16^. These experiments showed that MSRC is efficient for tumor classification and can achieve high accuracy. Li *et al*. proposed a new classifier called the maxdenominator reweighted sparse representation-based classifier (MRSRC) for cancer classification^5^. These experiments showed the efficiency and robustness of MRSRC. All SRC-based methods model a classification problem to identify a sparse representation of test samples, whereas the L1 sparsity constraint represents a test sample as the linear combination of these training samples.

In the sparse representation model, the test sample y ∈ R^*m*^ is used to represent a dictionary D = {*D*_1_, *D*_2_, … *D*_*c*_} ∈ *R*^*m*×*n*^, that is, y ≈ Dα where the sparse representation vector α ∈ *R*^*n*^ only shows several large entries. Then, the test samples are classified based on the solved vector αand the dictionary D. The selection of vector α and the dictionary D is crucial to the success of the sparse representation model. The previously described SRC-based methods directly regarded the training samples of all classes as the dictionary to represent the test sample and classified the test sample by evaluating which class leads to minimal reconstruction error. Although these methods showed interesting results, noise, outliers, incomplete measurements, and trivial information in the raw training data made this classification less effective. These naive methods also do not make maximize the discriminative information in the training samples. These problems can be addressed by properly learning a discriminative dictionary.

In general, discriminative dictionary learning methods can be divided into two categories. In the first category, a dictionary shared by all classes is learned, whereas the representation coefficients are discriminative. Jiang *et al*. proposed that samples of the same class possesses similar sparse representation coefficients^21^. Mairal *et al*. proposed a task-driven dictionary learning framework that minimizes the different risk functions of the representation coefficients for different tasks^22^. In general, these of methods aims to learn a shared dictionary by all classes and classify test samples with representation coefficients. However, the shared dictionary loses the class labels of the dictionary atoms. Thus, classifying the test samples based on the class-specific representation residuals is not feasible.

In the second category, discriminative dictionary learning methods learn a dictionary class by class, and atoms of the dictionary correspond to the subject class labels. Yang *et al*. learned a dictionary for each class, classified the test samples by using the representation residual, and applied dictionary learning methods to face recognition and signal clustering^23^. Wang *et al*. proposed a class-specific dictionary learning method for sparse modeling in action recognition^24^. In the previously mentioned methods, test samples are classified by using the representation residual associated with each class, but the representation coefficients are not used and are not enforced to be discriminative in the final classification.

To solve the previously discussed problems, Yang *et al*. proposed a Fisher discrimination dictionary learning framework to learn a structured dictionary^25^. In discrimination dictionary learning, the sparse representation coefficients present large between-class scatter and small within-class scatter. Each class-specific sub-dictionary presents good reconstruction of the training samples from that class and poor reconstruction of the other classes. By Fisher discrimination dictionary learning, the representation residual associated with each class can effectively be used for classification and the discrimination of representation coefficients can be exploited.

All SRC-based methods assume that the coding residual follows a Gaussian or Laplacian distribution, which may not be effective for describing the coding residual in practical GEP datasets. To address this problem, Yang *et al*. proposed a regularized robust coding (RRC) method for face recognition^26^. The RRC model searches for a maximum a posteriori (MAP) solution of the coding problem by assuming that the coding residual and representation coefficient are independent and identically distributed. However, either SRC-based or RRC methods or both do not take full advantage of discriminative information in representation coefficients. In the present study, we present RRC based on the Fisher discrimination dictionary learning method, a novel and effective cancer classification technique combining RRC methods and the concept of Fisher discrimination dictionary learning, which can maximize the use of discriminative information in representation coefficients and representation residuals. The proposed Fisher discrimination regularized robust coding (FDRRC) model extensively applies to various tumor GEP datasets and shows superior performance to different state-of-the-art SRC-based and machine learning-based methods in a variety of classification tasks.

The remainder of the paper is organized as follows: Section 2 mainly describes the experimental process and presents the experimental results obtained from eight tumor datasets. Section 3 discusses the proposed method, concludes the paper and outlines future studies. Section 4 describes the fundamentals of FDRRC.

## Results

In present study, eight publicly available tumor data sets are used to evaluate the performance of FDRRC. The experiment is divided into four sections. In the first section, cancer datasets and dataset preprocessing are introduced. In the second section, parameter selection is discussed. In the third section, describes the various samples used in the experiment with 400 top genes on eight datasets. In the fourth section, to make a fair performance comparison, cross-validation (CV) is presented. The proposed method is compared with several representative methods, such as SRC^18^, SVD + MSRC^27^ and MRSRC^5^. SRC, MSRC, and MRSRC are SRC-based methods that have been widely used in tumor classification in recent years. All experiments are implemented in the Matlab environment and conducted on a personal computer (Intel Core dual-core CPU with 2.93 GHz and 8 G RAM).

### Cancer datasets and dataset preprocessing

For a more comprehensive comparison of the performance of these methods, eight tumor GEP datasets are used to evaluate the proposed method. These datasets include five two-class datasets and three multi-class datasets. The summarized descriptions of the eight GEP datasets are provided in Table 1.

**Table 1:** The descriptions of eight data sets of tumor.

Data set	Classes	Genes	The number of samples
Acute leukemia data	2	7,129	72
Colon cancer data	2	2,000	62
Gliomas data	2	1,2625	50
DLBCL data	2	7,129	77
Prostate data	2	12,600	136
ALL data	6	12,625	248
MLLLeukemia data	3	12,582	72
LukemiaGloub data	3	7,129	72

The five two-class tumor datasets are acute leukemia dataset^28^, colon cancer dataset^29^, gliomas dataset^30^, diffuse large B-cell lymphoma (DLBCL) dataset^31^ and Prostate dataset^32^. The acute leukemia set contains 72 samples from two subclass. The colon cancer data set includes 62 samples, with gene expression data for 40 tumor and 22 normal colon tissue samples. The gliomas data set consists of 50 samples from two subclasses (glioblastomas and anaplastic oligodendrogliomas), and each sample contains 12,625 genes. For the DLBCL data set, RNA was hybridized to high-density oligonucleotide microarrays to measure the gene expression. The target dataset contains 77 samples of 7,129 genes. The target class has 2 states, including 58 diffuse large b-cell lymphoma samples and 19 follicular lymphoma samples. For the prostate tumor data set, the gene expression profiles were derived from tumors and non-tumor samples from prostate cancer patients, including 59 normal and 75 tumor samples. The number of genes is 12,600. Table 1 provides the details of the data sets.

For multi-class datasets, the data sets include the small round blue cell tumors (ALL)^33^, MLLLeukemia^34^, and LukemiaGloub^28^. The ALL data set total contains 248 samples and 12,626 genes from six subclasses. The MLLLeukemia data set contains 72 samples and 12,582 genes per sample with three subclasses. The LukemiaGloub data set contains 72 samples with three subclasses. Each sample contains 7,129 genes. Table 4 provides details of the data sets.

GEP data offer high dimensionality and a small sample size. Redundant and irrelevant data significantly affects classification. To compare the performance of FDRRC and SRC-based methods in the gene selection, the ReliefF algorithm is applied to the training set^35^. Then, the top 400 genes are selected from each dataset, thereby presenting a good trade-off between computational complexity and biological significance.

### Parameter selection

Five parameters should be set in the FDRRC model. The dictionary learning phase employs two parameters: *λ*_1_ and *λ*_2_, which are both presented in Eq.(8). In general, we search *λ*_1_, *λ*_2_ from a small set {0.001, 0.005, 0.01, 0.05, 0.1} by five-fold CV. The classifying phase includes three parameters, namely, μ and δ from the weight function Eq. (21) and w from residual function Eq. (24). Parameter μ controls the decreased rate of the weight *w*_*i*,*i*_; we can simply set μ = s/δ, where s = 8 is a constant. Parameter δ controls the location of the demarcation point, which can be obtained by using the following formula:

1 $${\rm{\delta }}={\rm{\pi }}{({\rm{e}})}_{\phi },$$

where π(e)_*φ*_ is the *φ*^*th*^ largest element of the set $\{{e}_{j}^{2},\,j=1,\,2,\,\cdots ,\,m\}$ and *φ* = ς(τm) outputs the largest integer smaller than τm. According to the experiments^7^, τ = 0.9 can be set in the classification of tumors. Parameter w can balance the contributions of the representation residual and representation vector to the classification. We search for w from a small set {0.0001, 0.0005, 0.001, 0.005, 0.01, 0.05, 0.1} by five-fold VC.

### Comparison of the balance division performance

Different divisions of the training set and test set can greatly affect the classification performance. To avoid the effects of an imbalanced training set, the balance division method (BDM) is designed to divide each original data set into a balanced training set and test set. For this BDM, Q samples from each subclass are randomly selected for use in the training set, and the remaining samples are used in the test set. Here, Q is an integer number. In the present study, we set Q = 5to $\min (|{c}_{i}|)-1$ samples per subclass as the training set and used the remaining samples for testing to guarantee that at least one sample in each category can be used in the test. Q denotes the number of training samples per class, and min(|*c*_*i*_|) denotes the minimum number of subclass set of samples in the training data. Suggesting that when Qis 5, then 5 samples per-subclass are randomly selected and used as the training set and the rest are assigned to the test set. In this experiment, the training/testing is performed 10 times, and the average classification accuracies are presented.

**Figure 1:**
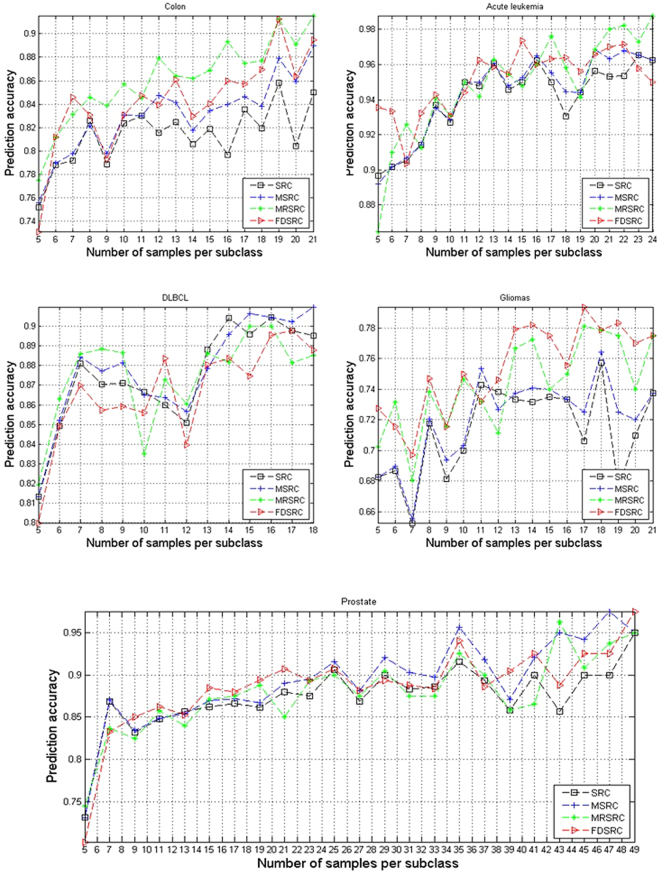
Comparison of prediction accuracy on five two-class classification datasets by varying the number of samples from per subclass.

**Figure 2:**
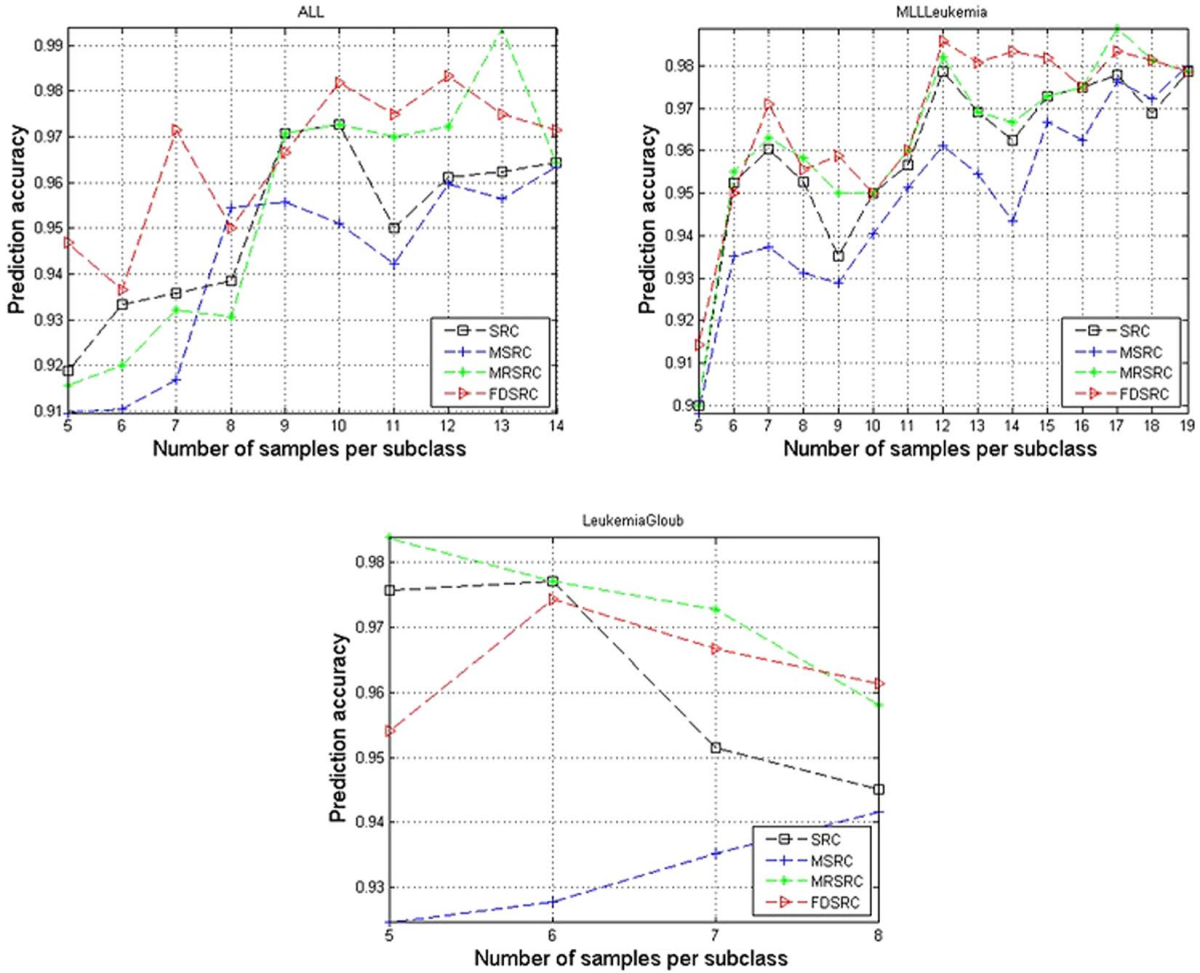
Comparison of prediction accuracy on three multi-class classification datasets by varying the number of samples from per subclass.

The average prediction accuracies that vary with different values of Q are shown in Figs 1 and 2, showing that, in the case of two-class classification, FDRRC achieves the highest classification accuracy in most cases in the acute leukemia and Gliomas datasets. Although gliomas are difficult to classify, FDRRC can still achieve the highest classification accuracy when Q = 17 samples per subclass are used in training. For the prostate dataset, FDRRC achieves the highest classification accuracy in most cases when the samples are few per subclass. In the case of multi-class classification, the experimental results indicate that FDRRC obtains a significant advantage in the ALL and MLLLeukemia datasets. Generally, the present methods are superior to other SRC-based methods in prediction accuracy not only on the four two-class classification datasets but also on the three multi-class classification datasets.

### Comparison with different numbers of genes

**Figure 3:**
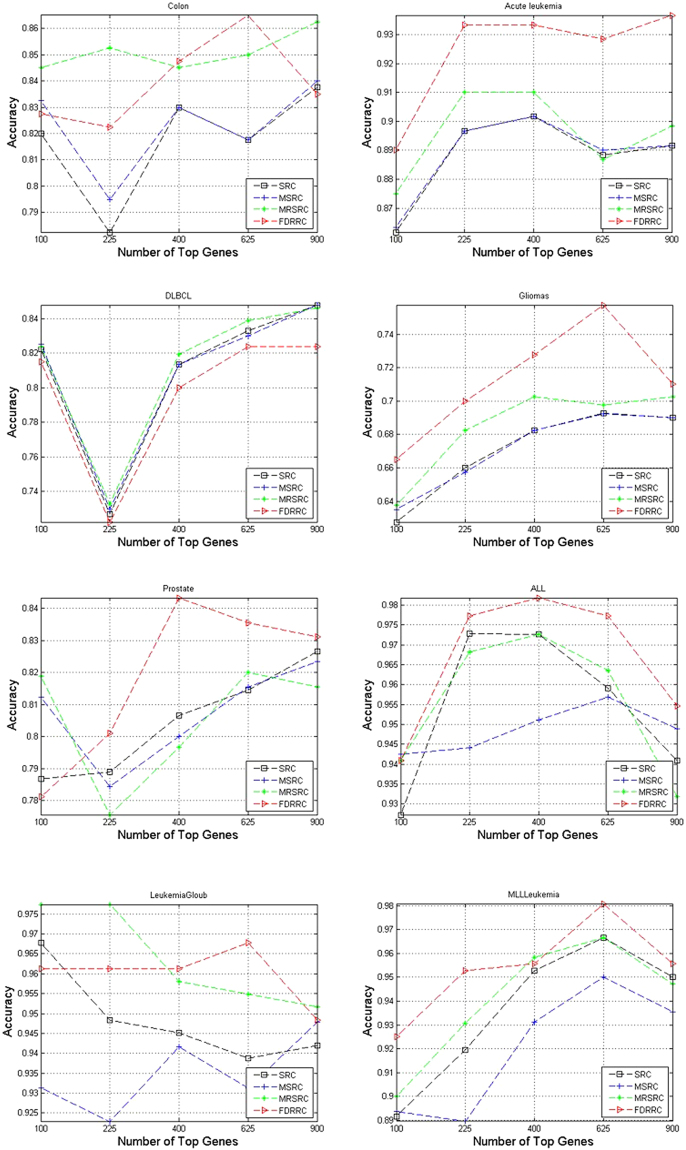
Comparison of accuracy on eight datasets by varying the number of top selected genes.

To compare the performance of the four models with different feature dimensions on eight tumor data sets, we run experiments using the ReliefF algorithm to select genes from 10^2^ to 30^2^ in increments of 5. For these experiments, the number of samples per subclass of the training set, was selected from {5, 6, 7, 8, 9, 10} by five-fold VC. The results are shown in Fig. 3.

Figure 3 presents the average prediction accuracy for the classification of eight tumor data sets. As shown in Fig. 3, FDRRC achieves the best accuracy in the five data sets in most cases, illustrating that FDRRC is robust with respect to the number of top genes. For Colon, Acute leukemia, DLBCL, Gliomas, Prostate and MLLLeukemia data sets, the accuracy of the curve increases with the increasing number of genes selected. Clearly, the selection of the top genes can improve the performance of all classification methods. For Acute leukemia dataset and ALL dataset, the best number of top genes is 400. These results suggest that the selection of the top 400 genes is reasonable.

### Comparison of 10-fold CV performance

To evaluate the classification performance on imbalanced split training/testing sets, we perform a 10-fold stratified CV experiment to evaluate the classification performance between FDRRC and SRC-based methods. All samples are randomly divided into 10 subsets and nine subsets are used for training, the remaining samples are used for testing.

The 10-fold CV results are summarized in Tables 2, 3 and 4. Table 2 shows that FDRRC achieves the highest level of accuracy in seven datasets. Particularly in multi-class datasets, FDRRC exhibits the best classification accuracy in all datasets. Table 3 indicates that FDRRC achieves the highest prediction sensitivity in six datasets, whereas FDRRC shows the best classification sensitivity in four tow-class datasets. Table 4 shows that FDRRC exhibits the highest specificity in seven datasets. Particularly in multi-class datasets, FDRRC exhibits the best classification accuracy in all datasets. Thus, we concluded that the excellent applicability of FDRRC whether in two-class or multi-class datasets, exhibits the best classification accuracy, the best classification sensitivity, and the best classification specificity in most cases.

**Table 2:** 10-fold CV prediction accuracy of eight tumor microarray datasets by using various classification methods with the top 400 genes.

Dataset	SRC	MSRC	MRSRC	FDRRC
Colon cancer data	77.42	80.65	82.26	**83**.**87**
Acute leukemia data	94.44	95.83	95.83	**98**.**61**
Gliomas data	70.00	70.00	74.00	**82**.**00**
DLBCL data	90.91	92.21	89.61	**96**.**10**
Prostate data	88.24	95.10	**96**.**08**	92.16
ALL data	97.18	97.58	**97**.**98**	**97**.**98**
MLLLeukemia data	97.22	**98**.**61**	**98**.**61**	**98**.**61**
LukemiaGloub data	94.44	95.83	97.22	**100**

**Table 3:** 10-fold CV prediction sensitivity of eight tumor microarray datasets by using various classification methods with the top 400 genes.

Dataset	SRC	MSRC	MRSRC	FDRRC
Colon cancer data	68.18	68.18	**81**.**82**	77.27
Acute leukemia data	92.00	92.00	88.00	**96**.**00**
Gliomas data	71.43	71.43	71.43	**82**.**14**
DLBCL data	94.74	94.74	**100**	**100**
Prostate data	92.31	94.23	**96**.**15**	**96**.**15**
ALL data	80.00	86.67	**93**.**33**	86.67
MLLLeukemia data	95.83	**100**	**100**	**100**
LukemiaGloub data	88.89	88.89	88.89	**100**

**Table 4:** 10-fold CV prediction specificity of eight tumor microarray datasets by using various classification methods with the top 400 genes.

Dataset	SRC	MSRC	MRSRC	FDRRC
Colon cancer data	82.50	**87**.**50**	82.50	**87**.**50**
Acute leukemia data	95.74	97.87	**100**	**100**
Gliomas data	68.18	68.18	77.27	**81**.**82**
DLBCL data	89.66	91.38	86.21	**94**.**83**
Prostate data	84.00	**96**.**00**	**96**.**00**	88.00
ALL data	99.14	98.71	98.71	**99**.**57**
MLLLeukemia data	**100**	**100**	**100**	**100**
LukemiaGloub data	**100**	**100**	**100**	**100**

## Discussions

The results of the present study, show that FDRRC outperforms the sparse representation-based methods (such as SRC, MSRC, and MRSRC) in most experiments. FDRRC outperforms the sparse representation-based methods probably because the representation residual associated with each class can be effectively used for classification, the discrimination of representation coefficients has been exploited, the coding residual is independent and identically distributed and the local center can help to distinguish outliers.

In the present, we proposed a new method, called FDRRC for classifying tumors. This method adopts the Fisher discrimination dictionary learning method and the concept of the local center with the RRC model. The FDRRC model learns a discriminative dictionary and seeks a MAP solution to the coding problem. Classification is achieved by a local center classifier, which takes full discriminative information in representation coefficients. We also compare the performance of FDRRC with those of three sparse representation-based methods by using eight tumor expression datasets. The results demonstrate the superiority of FDRRC and validate the effectiveness and efficiency of FDRRC in tumor classification.

Compared with the other methods, FDRRC exhibits a stable performance with respect to various datasets. The properties of this FDRRC algorithm should be further investigated. Thus, we will extend the algorithm with a superior discriminative dictionary and consider the driver genes to tailor the algorithm in our future studies. In addition, FDRRC will be used to predict miRNA^36^ and lncRNA-disease association^37^ in future studies.

## Methods

### Description of SRC problem

Assuming that X = {*X*_1_, *X*_2_, …, *X*_*c*_} ∈ *R*^*m*×*n*^ is a training sample set, where c corresponds to the number of subclasses, and m, n are dimensionality and the number of samples, respectively. The *j*_*th*_ class training samples *X*_*j*_ can be presented as columns of a matrix ${X}_{j}=[{x}_{j,1},\,{x}_{j,2},\,\cdots \,{x}_{j,{n}_{j}}]\in {R}^{m\times n},\,j=1,\,2,\,\cdots ,\,c$ where *x*_*j*,*i*_ is a sample of *j*_*th*_ class, and *n*_*j*_ refers to the number of *j*_*th*_ class training samples. Let L = {*l*_1_, *l*_2_, … *l*_*c*_} denote the label set, whereas y ∈ *R*^*m*^ is a test sample. Then, the SRC-based problem can be represented as follows:

2 $${\alpha }^{\wedge }=argmi{n}_{\alpha }\{{\Vert y-X\alpha \Vert }_{2}^{2}+\gamma {\Vert \alpha \Vert }_{1}\}$$

where ${\alpha }^{\wedge }=[{\alpha }_{1}^{\wedge },\,{\alpha }_{2}^{\wedge },\,\cdots ,\,{\alpha }_{c}^{\wedge }]$ includes the sparse representation coefficient of y with respect to X, and γ is a small positive constant. By obtaining representation coefficient *α*^∧^, SRC-based method assigns a label to test sample y according to the following equation:

3 $${e}_{i}={\Vert y-{X}_{i}{\alpha }_{i}^{\wedge }\Vert }_{2}^{2}$$

where ${\alpha }_{i}^{\wedge }$ is the sparse representation coefficient sub-vector associated with subclass *X*_*i*_. The classification rule is set as *identity*(*y*) = *argmin*_*i*_{*e*_*i*_}.

### Fisher Discrimination Dictionary Learning

Given the training samples X = {*X*_1_, *X*_2_, …, *X*_*c*_}, the Fisher discrimination dictionary learning model not only requires that D should be highly capable of representing X (i.e., X ≈ Dα) but also that D can strongly distinguish the samples in X. The Fisher discrimination dictionary learning model can be expressed as follows:

4 $${J}_{(D,X)}=argmi{n}_{(D,X)}\{r(X,D,\alpha )+{\lambda }_{1}{\Vert \alpha \Vert }_{1}+{\lambda }_{2}f(\alpha )\,s.t.\,{\Vert {d}_{n}\Vert }_{2}=1,\,\forall \,n\}$$

where *f*(*α*) is a discrimination term imposed on the coefficient matrix α, ${\Vert a\Vert }_{1}$ is the sparsity penalty, *r*(*X*, *D*, *α*)is the discriminative data fidelity term, and *λ*_1_ and *λ*_2_ are scalar parameters.

We can write *α*_*i*_ as ${\alpha }_{i}=[{\alpha }_{i}^{1};\,\cdots ;\,{\alpha }_{i}^{j};\,\cdots ;\,{\alpha }_{i}^{c}]$, where ${\alpha }_{i}^{j}$ is the representation coefficient of *X*_*i*_ over *D*_*i*_. For the discriminative data fidelity term *r*(*X*, *D*, *α*), *X*_*i*_ could be well represented by *D*_*i*_ but not by *D*_*j*,_*j* ≠ *i*. This relationship indicates that ${\alpha }_{i}^{i}$ should present several significant coefficients to achieve a small ${\Vert {X}_{i}-{D}_{i}{\alpha }_{i}^{i}\Vert }_{F}^{2}$, whereas ${\alpha }_{i}^{j},j\ne i$ should include small coefficients so that ${\Vert {D}_{i}{\alpha }_{i}^{i}\Vert }_{F}^{2}$ is small. Thus, the discriminative data fidelity term can be defined as follows:

5 $$r({X}_{i},D,{\alpha }_{i})={\Vert {X}_{i}-D{\alpha }_{i}\Vert }_{F}^{2}+{\Vert {X}_{i}-{D}_{i}{\alpha }_{i}^{i}\Vert }_{F}^{2}+\sum _{\begin{array}{c}j=1\\ j\ne i\end{array}}^{c}{\Vert {D}_{j}{\alpha }_{i}^{j}\Vert }_{F}^{2}.$$

For the discriminative coefficient term *f*(*α*), the Fisher discrimination criterion^38^ is expected to minimize the within-class scatter of α, denoted by *SW*(*α*), and maximize the between-class scatter of α, denoted by *SB*(*α*). *SW*(*α*) and *SB*(*α*) are defined as follows:

6 $${\rm{SW}}({\rm{\alpha }})=\sum _{i=1}^{c}{\sum }_{{\alpha }_{c}\in {\alpha }_{i}}({\alpha }_{c}-{m}_{i}){({\alpha }_{k}-{m}_{i})}^{T}\,and\,SB(\alpha )=\sum _{i=1}^{c}{n}_{i}({m}_{c}-m){({m}_{i}-m)}^{T},$$

where *m*_*i*_ and *m* are the mean vectors of *α*_*i*_ and α, respectively, and *n*_*i*_ is the number of samples in class *X*_*i*_. Thus, the criminative coefficient term can be defined as follows:

7 $$f(\alpha )=tr({\rm{SW}}({\rm{\alpha }}))-tr({\rm{SB}}({\rm{\alpha }}))+\eta {\Vert \alpha \Vert }_{F}^{2}$$

where *tr*(⋅) means the trace of a matrix, *η* is a parameter, and ${\Vert \alpha \Vert }_{F}^{2}$ is an elastic term.

Finally, the Fisher discrimination dictionary learning model can be expressed as follows:

8 $$mi{n}_{(D,X)}\{\sum _{i=1}^{c}r({X}_{i},D,{\alpha }_{i})+{\lambda }_{1}{\Vert \alpha \Vert }_{1}+{\lambda }_{2}(tr({\rm{SW}}({\rm{\alpha }}))-tr({\rm{SB}}({\rm{\alpha }})))+\eta {\Vert \alpha \Vert }_{F}^{2}\}s.t.\,{\Vert {d}_{n}\Vert }_{2}=1,\,\forall \,n$$

Optimization of the Fisher discrimination dictionary learning model can be divided into sub-problems, that is, updating α with a fixed D and updating D with a fixed α.

When α is updated, the dictionary D is fixed and can compute *α*_*i*_ class by class. When computing *α*_*i*_, all *α*_*j*_, *j ≠ i* are fixed. The objective function expressed in Eq. (8) is reduced to a sparse representation problem and can be written as follows:

9 $$mi{n}_{{\alpha }_{i}}\{r({X}_{i},D,{\alpha }_{i})+{\lambda }_{1}{\Vert {\alpha }_{i}\Vert }_{1}+{\lambda }_{2}{f}_{i}({\alpha }_{i})\}$$

with

$${f}_{i}({\alpha }_{i})={\Vert {\alpha }_{i}-M\Vert }_{F}^{2}-\sum _{k=1}^{c}{\Vert {M}_{k}-M\Vert }_{F}^{2}+\eta {\Vert {\alpha }_{i}\Vert }_{F}^{2},$$

where *M*_*k*_ and *M* are the mean vector matrices of class k and all classes, respectively. In this study, we set *η* = 1 for simplicity. Notably, all terms in Eq. (9), except for ${\Vert a\Vert }_{1}$, are differentiable. We rewrite Eq. (9) as follows:

10 $$mi{n}_{{\alpha }_{i}}\{Q({\alpha }_{i})+2\tau {\Vert {\alpha }_{i}\Vert }_{1}\},$$

where Q(*α*_*i*_) = *r*(*X*_*i*_, *D*, *α*_*i*_) + *λ*_2_ *f*_*i*_(*α*_*i*_) and τ = *λ*_1_/2. The method of FISTA^39^ can be employed to solve Eq. (10), as described in Table 5.

**Table 5:** Update of representation coefficient ${\rm{\alpha }}$ in the Fisher discrimination dictionary learning model.

**Input**: $${\boldsymbol{\sigma }}{\boldsymbol{,}}\,{\boldsymbol{\tau }}{\boldsymbol{ > }}{\boldsymbol{0}}{\boldsymbol{.}}$$
1. Initialization: $${\alpha }_{i}^{\wedge (1)}=0$$ and h = 1.
2. while convergence or the maximal itertion number is not reached do h + h = 1
$${\alpha }_{i}^{\wedge (h)}={S}_{\tau /\sigma }({\alpha }_{i}^{\wedge (h-1)}-\frac{1}{2\sigma }\nabla Q({\alpha }_{i}^{\wedge (h-1)}))$$
where $\nabla Q({\alpha }_{i}^{\wedge (h-1)})$ is the derivative of Q(*α*_*i*_) w.r.t ${\alpha }_{i}^{\wedge (h-1)}$, and *S*_*τ*/*σ*_ is a component-wise soft thresholding operator defined by Wright *et al*.^42^.
$${[{S}_{\tau /\sigma }(\alpha )]}_{j}=\{\begin{array}{cc}0 & |{\alpha }_{j}|\le \tau /\sigma \\ {\alpha }_{j}-sign({\alpha }_{j})\tau /\sigma & otherwise\end{array}$$
3. Return $${\alpha }_{i}^{\wedge }={\alpha }_{i}^{\wedge (h)}$$.

When updating D = [*D*_1_, *D*_2_, …, *D*_*c*_], the coefficient α is fixed. We also update ${D}_{i}=[{d}_{1},{d}_{2},\,\cdots ,\,{d}_{{n}_{i}}]$ class by class. When updating *D*_*i*_, all *D*_*j*_, *j* ≠ *i*, are fixed. The objective function expressed in Eq. (8) is reduced to:

11 $$mi{n}_{{D}_{i}}\{{\Vert {X}^{\sim }-{D}_{i}{\alpha }^{i}\Vert }_{F}^{2}+{\Vert {X}_{i}-{D}_{i}{\alpha }_{i}^{i}\Vert }_{F}^{2}+\sum _{j=1,j\ne i}^{c}{\Vert {D}_{i}{\alpha }_{j}^{i}\Vert }_{F}^{2}\}\,s.t.\,\Vert {d}_{l}\Vert =1,\,l=1,2,\,\cdots ,\,{n}_{i}$$

where ${X}^{\sim }=X-{\sum }_{j=1,j\ne i}^{c}\,{D}_{j}{\alpha }^{j}$ and *α*^*j*^ is the representation matrix of *X* over *D*_*i*_. Eq. (11) could be re-written as follows:

12 $$mi{n}_{{D}_{i}}{\Vert {{\rm{\Lambda }}}_{i}-{D}_{i}{Z}_{i}\Vert }_{F}^{2}\,s.t.\,{\Vert {d}_{l}\Vert }_{2}=1,l=1,2,\,\cdots ,\,{n}_{i}$$

where Λ_*i*_ = [*X*^~^ *X*_*i*_0 … 00 … 0], ${Z}_{i}={\alpha }^{i}{\alpha }_{i}^{i}{\alpha }_{1}^{i}\cdots {\alpha }_{i-1}^{i}{\alpha }_{i+1}^{i}\cdots {\alpha }_{c}^{i}$ and 0 is a zero matrix with the appropriate size based on the context. Eq. (12) can be efficiently solved by updating each dictionary atom one by one via the algorithm of Yang *et al*.^40^. The update of dictionary D is described in Table 6.

**Table 6:** Update of dictionary D in the Fisher discrimination dictionary learning model.

Fix α and update each ${D}_{i},\,i=1,\,2,\,\mathrm{...}\,C$, by solving Eq. (12)
1. Let ${Z}_{i}=[{z}_{1};\,{z}_{2};\,\mathrm{...};\,{z}_{{n}_{i}}]$ and ${D}_{i}=[{d}_{1},\,{d}_{2},\,\mathrm{...}\,{d}_{{n}_{i}}]$, where *z*_*j*_, *j* = 1, 2, ... *n*_*i*_ is the row vector of z_*i*_, and *d*_*j*_ is the *j*_*th*_ column vector of *D*_*i*_.
2. Fix all ${d}_{j},\,l\ne j$, update *d*_*j*_. Let $Y={{\rm{\Lambda }}}_{i}-\sum _{l\ne j}{d}_{l}{z}_{l}$. The minimization of Eq. (12) becomes
$${{\rm{\min }}}_{{d}_{j}}{\Vert Y-{d}_{j}{z}_{j}\Vert }_{F}^{2}s.t.{\Vert {d}_{j}\Vert }_{2}=1$$
After some deviation, we could get the solution ${d}_{j}=Y{z}_{j}^{T}/{\Vert Y{z}_{j}^{T}\Vert }_{2}$.
3. Then Fix D and update α like Table 5.

### Description of RRC

In the SRC-based method, coding residual e = y − Dα follows Gaussian distribution^25^. However, in practice, Gaussian priors on e may be invalid, especially when GEP data are corrupted and contain outliers. To deal with this problem, we can consider tumor classification from the view point of Bayesian estimation, especially MAP estimation. Based on MAP estimation, sparse representation coefficient α can be expressed as follows^26^:

13 $${\alpha }^{\wedge }=argma{x}_{\alpha }\,\mathrm{ln}\,p(\alpha |y)$$

Then, by using Bayesian formulation, we can obtain the following:

14 $${\alpha }^{\wedge }=argma{x}_{\alpha }\{\mathrm{ln}\,p(y|\alpha )+\,\mathrm{ln}\,p(\alpha )\}$$

Assuming that elements *e*_*i*_ of coding residual e = y − Dα = [*e*_1_; *e*_2_; … *e*_*m*_] are independent and identically distributed and feature the probability density function (PDF) *f*_*θ*_(*e*_*i*_), then we can obtain the equation below:

15 $$\mathrm{ln}\,p(y|\alpha )=\prod _{i=1}^{m}{f}_{\theta }({y}_{i}-{r}_{i}\alpha )$$

Meanwhile, assuming that element *α*_*i*_ of sparse representation coefficient α = [*α*_1_; *α*_2_; …; *α*_*n*_] are independent and identically distributed and contain the PDF *f*_*σ*_(*α*_*i*_), then we can acquire the following formula:

16 $${\rm{p}}({\rm{\alpha }})=\prod _{j=1}^{n}{f}_{\sigma }({\alpha }_{j})$$

Finally, MAP estimation of α can be expressed as follows:

17 $${\alpha }^{\wedge }=argma{x}_{\alpha }\{\prod _{i=1}^{m}{f}_{\theta }({y}_{i}-{r}_{i}\alpha )+\prod _{j=1}^{n}{f}_{\sigma }({\alpha }_{j})\,\}$$

Letting ${\rho }_{\theta }(e)=-\,\mathrm{ln}\,{f}_{\theta }(e)$ and _*ρσ*_(*α*) = −*lnf*_*σ*_(*α*), then, the above equation can be converted into the following:

18 $${\alpha }^{\wedge }=argma{x}_{\alpha }\{\sum _{i=1}^{m}{\rho }_{\theta }({y}_{i}-{r}_{i}\alpha )+\sum _{j=1}^{n}{\rho }_{\sigma }({\alpha }_{j})\}$$

The above model is called RRC. Two key issues must be considered to solve the RRC model: determining distributions of *ρ*_*θ*_(*e*) and *ρ*_*σ*_(*α*); and minimizing energy function.

For *ρ*_*θ*_(*e*), given diversity in gene variations, predefining distribution presents difficulty. In RRC model, unknown PDF *ρ*_*θ*_(*e*) is assumed symmetric, differentiable, and monotonic. Therefore, *ρ*_*θ*_(*e*) features the following properties: (1) *ρ*_*θ*_(0) is global minimal of *ρ*_*θ*_(*Z*); (2) *ρ*_*θ*_(*Z*) = *ρ*_*θ*_(−*Z*); (3) if |*Z*_1_| < |*Z*_2_|, then *ρ*_*θ*_(*Z*_1_) < *ρ*_*θ*_(*Z*_2_). Without loss of generality, we let *ρ*_*θ*_(0) = 0. Meanwhile, *ρ*_*θ*_(*e*) is allowed to feature a more flexible shape, which adapts to input testing sample y, to make the system more robust to outliers. Then, by Taylor expansion, Equation (18) can be approximated as follows:

19 $${\alpha }^{\wedge }=argma{x}_{\alpha }\{\frac{1}{2}{\Vert {W}^{1/2}(y-D\alpha )\Vert }_{2}^{2}+\sum _{j=1}^{n}{\rho }_{\sigma }({\alpha }_{j})\}$$

where *W* is a diagonal matrix and can be updated via the following formula:

20 $${W}_{i,i}={\omega }_{\theta }({e}_{0,i})=\rho {^{\prime} }_{\theta }({e}_{0,i})/{e}_{0,i}$$

Thus, minimization of RRC focuses on calculating diagonal weight matrix *W*. As *ρ*_*θ*_(*e*) is symmetric, differentiable, and monotonic, *ω*_*θ*_(*e*_*i*_) can be assumed as continuous and symmetric while being inversely proportional to *e*_*i*_. With these considerations, the logistic function which features the same properties is a good choice for *ω*_*θ*_(*e*_*i*_)^41^. Thus, we can obtain the following:

21 $${\omega }_{\theta }({e}_{i})=\exp (-\mu {e}_{i}^{2}+\mu \delta )/(1+\exp (-\mu {e}_{i}^{2}+\mu \delta ))$$

where parameters μ and δ represent two positive scalars. Parameter μcontrols decreasing rate from 1 to 0, and δ controls location of demarcation point. With Equations (20) and (21) and *ρ*_*θ*_(0) = 0, we can formulate Equation (22):

22 $${\rho }_{\theta }({e}_{i})=-\frac{1}{2\mu }(\mathrm{ln}(1+\exp (-\mu {e}_{i}^{2}+\mu \delta ))-\,\mathrm{ln}(1+\exp (\mu \delta )))$$

For *ρ*_*σ*_(*α*), we can assume that sparse representation coefficient *α*_*i*_ follows a generalized Gaussian distribution as only the representation coefficients associated with training samples from the target class can feature high absolute values. As we do not know beforehand the class of the test sample, a reasonable prior can be that only a small percent of representation coefficients contains significant values. Then, we can used the following equation:

23 $${f}_{\sigma }({\alpha }_{j})=\beta exp\{-{(\frac{{\lfloor \alpha \rfloor }_{j}}{{\sigma }_{\alpha }})}^{\beta }\}/(2{\sigma }_{\alpha }{\rm{\Gamma }}(1/\beta ))$$

where Γ is the gamma function.

After determining distributions *ρ*_*θ*_(*e*) and *ρ*_*σ*_(*α*), minimized energy function can be used in the iteratively reweighted RRC (IR^3^C) algorithm, which was designed by Yang *et al*., to solve the RRC model efficiently^26^. The RRC (IR3C) algorithm is described in Table 7.

**Table 7:** The RRC algorithm.

1. Set the initial value of iteration count *t* = 1.
2. Compute the coding residual:
$${e}^{(t)}=y-D{\alpha }^{(t)}$$
where ${\alpha }^{({\rm{1}})}=[\frac{1}{m};\,\frac{1}{m};\,\cdot \cdot \cdot ;\,\frac{1}{m};]$ is the initial vector, and *m* is the mean of all training samples.
3. Estimate weight value of each gene as follows:
$${\omega }_{\theta }({{e}_{i}}^{(t)})={\rm{1}}/(1+\exp (-\mu {({e}_{i}^{(t)})}^{2}-\mu \delta ))$$
where *μ* and *δ* are estimated in each iteration, and *δ* is associated with residual.
4. Weighted regularized sparse representation coefficient:
$${\alpha }^{\ast }={{\rm{argmin}}}_{\alpha }\{\frac{1}{2}{\Vert {({w}^{(t)})}^{0.5}(y-D\alpha )\Vert }_{2}^{2}+\sum _{j=1}^{n}{\rho }_{\sigma }({\alpha }_{j})\}$$
where *w*^(*t*)^ is the estimated diagonal weight matrix with ${w}_{i,i}^{(t)}={\omega }_{\theta }({e}_{i}^{(t)},\,{\rho }_{\sigma }({\alpha }_{j})=\lambda {|{\alpha }_{j}|}^{\beta }{\rm{a}}{\rm{n}}{\rm{d}}\,\beta =1$.
5. Update the sparse representation coefficients:
If *t* = 1, *α*^(*t*)^ = α^*^;
If *t* > 1, *α*^(*t*)^ = *α*^(*t*−1)^ + *υ*^(*t*)^(α^*^−*α*^(*t*−1)^);
where 0 < *υ*^(*t*)^ ≤ 1 is a suitable step size that can be search from 1 to 0 by the standard line-search process^43^.
6. Reconstruct the test sample by sparse representation coefficient and all metagenes:
$${y}_{rec}^{(t)}=D{\alpha }^{(t)}$$ and let *t* = *t* + 1.
7. Go back to Step 4 until condition of convergence ${\Vert {W}^{(t)}-{W}^{(t-1)}\Vert }_{2}/{\Vert {W}^{(t-1)}\Vert }_{2} < \phi$, where *φ* is a small positive scalar) is met, or maximal number of iterations is reached.

### Local center classifier

Equation (3) is the classification function of SRC-based methods that only consider discrimination capability of representation residuals and not the discrimination capability of representation vectors.

Assuming that *m*_*i*_ is the mean sparse representation coefficient vector of class *X*_*i*_, mean vector *m*_*i*_ can be viewed as the center of class *X*_*i*_ in the transformed space comprising D. Thus, we label *m*_*i*_ as the local center. For classification of tumor, when y originates from class *i*, residual ${\Vert y-{D}_{i}{\alpha }_{i}^{\wedge }\Vert }_{2}^{2}$should be small while ${\Vert y-{D}_{j}{\alpha }_{j}^{\wedge }\Vert }_{2}^{2},\,j\ne i$, should be big. In addition, sparse representation coefficient vector α^∧^ should be close to *m*_*i*_ but far from mean vectors of other classes. Considering the above factors, we define the following classifier:

24 $${e}_{i}={\Vert y-{D}_{i}{\alpha }_{i}^{\wedge }\Vert }_{2}^{2}+w{\Vert {{\rm{\alpha }}}^{\wedge }-{m}_{i}\Vert }_{2}^{2}$$

where *w* is a parameter for balancing contribution of the two terms to classification. Finally, we can obtain the label of y according to the following formula:

25 $$identity(y)={{\rm{argmin}}}_{i}({e}_{i})$$

### Algorithm of FDRRC

By combining the IR3C algorithm^26^ and Fisher discrimination dictionary learning model, we can obtain the algorithm of FDRRC. Table 8 shows the overall procedure of the algorithm.

**Table 8:** The FDRRC algorithm.

**Input**: Training samples *X* = [*X*_1_, *X*_2_, ..., *X*_*C*_] ∈ *R*^*m*×*n*^
Testing samples *y* ∈ *R*^*m*^
**Output**: Label *l* of *y*.
**1. Initialize** *D*.
We initialize the atoms of *D*_*i*_ as the eigenvectors of *X*_*i*_.
**2. Update coefficient** *α*.
Fix *D* and solve *α*_*i*_, *i* = 1, 2, ... *C*, one by one by solving Eq. (9) with the algorithm presented in Table 5.
**3. Update dictionary** *D*.
Fix *α* and update each ${D}_{i},\,i=1,2,\,\mathrm{...}\,C,$ by solving Eq. (12) with the algorithm presented in Table 6.
**4. Classify test sample** y.
Fix *α* and *D*, and solve the sparse representation *α*^∧^ of y with the algorithm presented in Table 7.
When the algorithm converges, we can classify the test samples as follows:
$$identity(y)={\rm{\arg }}\,{{\rm{\min }}}_{i}\{{\Vert {W}_{final}^{1/2}(y-{D}_{i}{\alpha }_{i}^{\wedge })\Vert }_{2}+wg{\Vert {\alpha }^{\wedge }-{m}_{i}\Vert }_{2}^{2}$$,
where *W*_*final*_ is the final weight matrix, ${\alpha }_{{\rm{i}}}^{\wedge }$ is the final sub- sparse representation coefficient vector associated with class *i*, and *α*^∧^ is the final representation coefficient vector.
